# A Missing PD-L1/PD-1 Coinhibition Regulates Diabetes Induction by Preproinsulin-Specific CD8 T-Cells in an Epitope-Specific Manner

**DOI:** 10.1371/journal.pone.0071746

**Published:** 2013-08-19

**Authors:** Cornelia Schuster, Helen Brosi, Katja Stifter, Bernhard O. Boehm, Reinhold Schirmbeck

**Affiliations:** Department of Internal Medicine I, Ulm University Hospital, Ulm, Germany; St. Vincent’s Institute, Australia

## Abstract

Coinhibitory PD-1/PD-L1 (B7-H1) interactions provide critical signals for the regulation of autoreactive T-cell responses. We established mouse models, expressing the costimulator molecule B7.1 (CD80) on pancreatic beta cells (RIP-B7.1 tg mice) or are deficient in coinhibitory PD-L1 or PD-1 molecules (PD-L1^−/−^ and PD-1^−/−^ mice), to study induction of preproinsulin (ppins)-specific CD8 T-cell responses and experimental autoimmune diabetes (EAD) by DNA-based immunization. RIP-B7.1 tg mice allowed us to identify two CD8 T-cell specificities: pCI/ppins DNA exclusively induced K^b^/A_12–21_-specific CD8 T-cells and EAD, whereas pCI/ppinsΔA_12–21_ DNA (encoding ppins without the COOH-terminal A_12–21_ epitope) elicited K^b^/B_22–29_-specific CD8 T-cells and EAD. Specific expression/processing of mutant ppinsΔA_12–21_ (but not ppins) in non-beta cells, targeted by intramuscular DNA-injection, thus facilitated induction of K^b^/B_22–29_-specific CD8 T-cells. The A_12–21_ epitope binds K^b^ molecules with a very low avidity as compared with B_22–29_. Interestingly, immunization of coinhibition-deficient PD-L1^−/−^ or PD-1^−/−^ mice with pCI/ppins induced K^b^/A_12–21_-monospecific CD8 T-cells and EAD but injections with pCI/ppinsΔA_12–21_ did neither recruit K^b^/B_22–29_-specific CD8 T-cells into the pancreatic target tissue nor induce EAD. PpinsΔA_12–21_/(K^b^/B_22–29_)-mediated EAD was efficiently restored in RIP-B7.1^+^/PD-L1^−/−^ mice, differing from PD-L1^−/−^ mice only in the tg B7.1 expression in beta cells. Alternatively, an ongoing beta cell destruction and tissue inflammation, initiated by ppins/(K^b^/A_12–21_)-specific CD8 T-cells in pCI/ppins+pCI/ppinsΔA_12–21_ co-immunized PD-L1^−/−^ mice, facilitated the expansion of ppinsΔA_12–21_/(K^b^/B_22–29_)-specific CD8 T-cells. CD8 T-cells specific for the high-affinity K^b^/B_22–29_- (but not the low-affinity K^b^/A_12–21_)-epitope thus require stimulatory ´help from beta cells or inflamed islets to expand in PD-L1-deficient mice. The new PD-1/PD-L1 diabetes models may be valuable tools to study under well controlled experimental conditions distinct hierarchies of autoreactive CD8 T-cell responses, which trigger the initial steps of beta cell destruction or emerge during the pathogenic progression of EAD.

## Introduction

Type 1 diabetes (T1D) is an autoimmune disorder, in which insulin-producing beta cells are destroyed by the cellular immune system [1], [2], [3]. Diabetes development is characterized by progressive infiltration of T-cells into the pancreatic islets and beta cell destruction, resulting in severe hyperglycemia. Disease in man is triggered by poorly defined antigens and factors that finally result in the breakdown of central and/or peripheral tolerance and activation of autoreactive CD4^+^ and/or CD8^+^ T-cells [1], [4]. There is increasing evidence from patients with T1D that autoreactive CD8^+^ T-cells are involved in the development of disease but it is difficult to detect these rare lymphocytes and to assign their individual effects during the progression of diabetes [5], [6], [7]. It is assumed that the nature of an autoantigen-derived peptide and its presentation by MHC class I molecules plays a central role in the development of T-cell-mediated autoimmunity [8]. In the NOD mouse model [9], the binding of insulin-derived self peptides to MHC class I or class II molecules is weak and caused by unfavoured binding registers [10], [11], [12]. This suggests that non-conventional antigenic epitope processing and presentation may contribute to the induction of autoreactive immune responses [7], [13].

Spontaneous diabetes development in the NOD mouse model elucidated many aspects of diabetogenic immune responses [9]. Furthermore, different mouse models have been used to characterize *de novo* induction of well-defined T-cell responses and their pathogenic cross-talk with beta cells, which selectively express transgene-encoded ‘neo-self’ antigens under rat insulin promoter (RIP) control [14]. We used transgenic (tg) RIP-B7.1 mice, expressing the costimulatory molecule B7.1 (CD80) on pancreatic beta cells [15], to characterize induction of preproinsulin (ppins)-specific CD8 T-cells and experimental autoimmune diabetes (EAD) by DNA-based immunization [16], [17], [18], [19]. A single injection of ppins-encoding DNA (pCI/ppins) efficiently induced CD8 T-cell-mediated EAD in both, male and female RIP-B7.1 tg mice with a median onset of 2–3 weeks post immunization and a cumulative diabetes incidence of >95% by week 4 [17]. In these mice, progressive invasion of insulin A-chain-derived K^b^/A_12–21_-specific CD8 T-cells into pancreatic islets precedes hyperglycemia and insulin deficiency. K^b^/A_12–21_-specific CD8 T-cells and EAD were efficiently induced by pCI/ppins in MHC class II-deficient (Aα^−/−^) RIP-B7.1 mice (RIP-B7.1^+^/MHC-II^−/−^) with no conventional CD4 T-cells and in RIP-B7.1 tg mice acutely depleted of CD4 T-cells with anti CD4 antibody [17], [18]. The RIP-B7.1 tg model hence provides an attractive experimental approach to study CD4 T-cell-independent induction of EAD by ppins-specific CD8 T-cells.

We further investigated the impact of coinhibitory ‘programmed death-1’ (PD-1)/‘programmed death-ligand-1’ (PD-L1 or B7-H1) molecules on the pathogenicity of ppins-specific CD8 T-cells [19]. PD-1/PD-L1 interactions provide critical inhibitory signals to T-cell responses [20], [21], [22] and facilitate establishment of self-tolerance in NOD mice [23], [24], [25], [26]. There is also evidence from human T1D patients that polymorphic PD-1 gene variations are associated with the susceptibility to disease [27], [28]. Immunization with pCI/ppins DNA efficiently primed K^b^/A_12–21_-specific CD8 T-cells and EAD in coinhibiton-deficient PD-L1^−/−^ and PD-1^−/−^ mice [19]. K^b^/A_12–21_-specific CD8 T-cells were also primed in wild type (wt) C57BL/6 (B6) mice but these cells revealed their diabetogenic potential only after treatment with anti PD-L1 antibody [19]. Furthermore, a deficiency of either PD-L1 in antigen presenting beta cells or PD-1 in T-cells was required to induce K^b^/A_12–21_-mediated EAD in bone marrow chimeric mice [19]. This suggested that PD-1/PD-L1-mediated signals regulate beta cell-destruction by K^b^/A_12–21_-specific CD8 T-cells.

During the course of EAD in RIP-B7.1 tg mice *ex vivo* stimulation of ppins-primed CD8 T-cells with the K^b^/A_12–21_ peptide, but not with all other peptides of a ppins-specific library, revealed a CD8 T-cell population with specifically inducible IFNγ expression [19]. This suggested that the K^b^/A_12–21_ is the only diabetogenic epitope in ppins-immune RIP-B7.1 tg mice. However, a mutant ppinsΔA_12–21_ antigen (with a deletion of the COOH-terminal A_12–21_ sequence) also induced CD8 T-cell-mediated EAD in RIP-B7.1 tg mice, indicating that EAD can be induced by CD8 T-cell responses that have specificities other than K^b^/A_12–21_ [18]. In this study, we mapped the alternative CD8 T-cell epitope in pCI/ppinsΔA_12–21_-immune RIP-B7.1 tg mice and investigated the antigen expression and processing requirements to prime this CD8 T-cell specificity and EAD. We further used coinhibiton-deficient PD-1^−/−^, PD-L1^−/−^ and RIP-B7.1^+^/PD-L1^−/−^ mice (differing from PD-L1^−/−^ mice only in the tg B7.1 expression in beta cells) to determine whether induction of ppins- and ppinsΔA_12–21_-specific CD8 T-cell responses and diabetes development depends on specific costimulatory and coinhibitory signals from pancreatic beta cells.

## Materials and Methods

### Ethics Statement

All mouse immunization studies were carried out in strict accordance with the recommendations in the Guide for the Care and Use of Laboratory Animals of the German Federal Animal Protection Law. The protocols were approved by the Committee on the Ethics of Animal Experiments of the University of Ulm (Tierforschungszentrum Ulm, Oberberghof) and the Regierungspräsidium Tübingen (Permit Numbers: 897 and 1105 to RS). All immunizations were performed under short time Isofluran anesthesia, and all efforts were made to minimize suffering.

### Mice

RIP-B7.1 mice were backcrossed for >15 generations to the C57BL/6 (H-2^b^) background as described [15]. C57BL/6 (B6) H-2^b^ mice were obtained from Janvier (Le Genets-St-Isle; France). PD-L1^−/−^ (B7-H1^−/−^ or CD274^−/−^) mice [29], PD-1^−/−^ mice [30], CD28^−/−^ mice (Jackson Laboratory, USA), MHC class II-deficient Aα^−/−^ mice [31] and RIP-B7.1 mice [15] were bred and kept under standard pathogen-free conditions in the animal colony of Ulm University (Ulm, Germany). We further generated RIP-B7.1 mice that were deficient for PD-L1 (RIP-B7.1^+^/PD-L1^−/−^) or MHC class II molecules (RIP-B7.1^+^/MHC-II^−/−^). Male and female mice were used in the experiments at 6–8 weeks of age.

### Construction of Expression Plasmids

The sequences of the different ppins antigens were codon-optimized and synthesized by GeneArt (Regensburg, Germany). All constructs were cloned into the pCI vector (cat.no. E1731, Promega, Mannheim, Germany) using the *NheI* and *NotI* restriction sites. Batches of plasmid DNA were produced in *E. coli* by PlasmidFactory GmbH (Bielefeld, Germany).

### Characterization of Antigen Expression

Human embryonal kidney cells (HEK-293 cells) were transiently transfected with the indicated plasmid DNAs using the calcium phosphate method. Cells were labeled with 100 µCi ^35^S-methionine/cysteine (cat. no. IS103, Hartmann Analytic GmbH) between 36 and 48 h post transfection and subsequently lysed with pH 8.0 lysis buffer (100 mM NaCl, 0.5% NP40 and 100 mM Tris-hydrochloride) supplemented with the protease inhibitors, leupeptin and aprotinin. Extracts were cleared by centrifugation and precipitated with polyclonal rabbit H-86 anti-insulin (cat. no. sc-9168, Santa Cruz Biotechnology) and protein G sepharose. Precipitates were processed for SDS-PAGE (15%) and subsequent fluorography of the gels. Alternatively, non-labeled cells were lysed in a SDS-containing buffer (3% SDS, 50 mM Tris-hydrochloride, 5% β-mercaptoethanol) and, for high resolution of protein bands, samples were directly loaded onto urea-containing SDS-polyacrylamide gels (16%) [32]. Gels were blotted onto a nitrocellulose membrane using the iBlot® Dry Blotting System (cat. no. IB3010-01; Invitrogen, Carlsbad, CA, USA). Membranes were blocked for 20 min at RT in a buffer supplemented with 0.1% Tween 20, 0.1% gelatine and 3% milk powder, followed by successive incubations with rabbit H-86 anti-insulin antibody and HRP-conjugated anti rabbit IgG (cat. No. NA9340; GE Healthcare, Chalfont St Giles, UK). Specific protein bands were detected using the Immobilon™ Western Chemoluminescent HRP substrate (cat. No. WBKLS0100; Millipore, Bedford, MA, USA) followed by subsequent exposure of the membranes to an Amersham Hyperfilm ECL (cat. No. 92004; GE Healthcare).

### Immunization of Mice

Plasmid DNA (75–100 µg/mouse) dissolved in PBS was injected into both tibialis anterior muscles. Diabetes was diagnosed if two consecutive blood glucose values (within 2 days) exceeded 250 mg/dl, i.e. 13.8 mmol/l (Disetronic Freestyle, Sulzbach, Germany).

### Histology

H&E staining and immunohistochemistry of pancreatic sections was performed as described previously [16]. For the staining of insulin, CD8^+^ or CD4^+^ cells the following primary antibodies were used: polyclonal guinea pig anti insulin serum (cat. No. A0564; Dako, Carpinteria, CA, USA), rat α-CD8 (cat. No. MCA2694; AbD Serotec, Oxford, UK) and rat α-CD4 (cat. No. MCA1767GA; AbD Serotec). These primary antibodies were detected with the secondary antibodies anti guinea pig IgG-FITC (cat. No. F-6261; Sigma-Aldrich, St Louis, MO, USA) and anti rat IgG-TRITC (cat. No. T4280; Sigma-Aldrich). Furthermore, sections were directly stained with PE-conjugated antibodies α-F4/80 (cat. No. 12-4801-80; eBioscience, Frankfurt, Germany) and α-CD11c (cat. No. 553802; BD Biosciences, Heidelberg, Germany). Sections were covered and mounted with Cytoseal60 mounting medium (cat. no. 18006, EMS). Finally, the images were captured with an Olympus IX71 fluorescence microscope equipped with a digital camera (C4742, Hamamatsu). Edition of the pictures was performed using ImageJ software (http://rsbweb.nih.gov/if/).

### Isolation of CD8 T-cells from Pancreatic Tissues

Pancreata were perfused *in situ* with collagenase P (cat. no. 11213865001, Roche) dissolved at 1 mg/ml in Hanks Balanced Salt Solution (HBSS), removed, digested again with collagenase P for 8 min at 37°C and washed twice with cold HBSS supplemented with 10% FCS. Pancreatic cells were purified with Histopaque-1077 (cat. no. 10771, Sigma-Aldrich) by centrifugation for 15 min at 2400 rpm.

### Determination of Specific CD8 T-cell Frequencies

To detect ppins-specific CD8 T-cell responses, we used a ppins-specific peptide library (*i.e.* 10mers with two amino acids offset) (JPT Peptide Technologies, Berlin, Germany). Peptides were dissolved in DMSO at a concentration of 10 mg/ml and diluted with culture medium prior to use. Pancreatic cells (10^5^/100 µl) were incubated for 16 h in Ultra Culture medium (cat. no.BE 12-725F, Lonza, Belgium) containing 10 µg/ml of the indicated peptides in the presence of brefeldin A (0.5 µg/ml) (cat. no. 15870; Sigma, Taufkirchen, Germany). Cells were harvested, surface stained with APC-conjugated anti CD8 antibody (cat. no. 17-0081-83, BD Biosciences, Heidelberg, Germany), fixed with 2% paraformaldehyde, resuspended in permeabilization buffer (HBSS, 0.5% BSA, 0.5% saponin, 0.05% sodium azide), and stained with FITC-conjugated anti-IFNγ antibody (cat. no.554411; BD Biosciences, Heidelberg, Germany). Non-specific binding of antibodies to Fc-receptor was blocked by preincubating cells with mAb 2.4G2 (cat. no. 01241D; BD Biosciences, Heidelberg, Germany) directed against the FcγRIII/II CD16/CD32 (0.5 µg mAb/10^6^ cells/100 µl). Frequencies of IFNγ^+^ CD8 T-cells were determined by flow cytometry (FCM) using a BD LSR-II Flow Cytometer.

Furthermore, specific CD8 T-cells were analyzed with K^b^/B_22–29_ tetramers (Glycotope, Heidelberg, Germany). Freshly isolated cells were washed twice in PBS/0.3% w/v BSA/0.1% w/v sodium azide. Non-specific binding of antibodies to Fc-receptor was blocked by preincubating cells with mAb 2.4G2 as described above. Cells were incubated for 30 min at 4°C with FITC-labeled anti-CD8 mAb (BD Biosciences, Heidelberg, Germany) and PE- or APC-conjugated tetramers. Cells were washed and analyzed by FCM.

### Statistics

The statistical significance of differences in the mean CD8 T-cell frequencies between groups was determined by the unpaired student’s t-test. The statistical significance of diabetes induction in immunized mice was determined by the log-rank test. Data were analyzed using PRISM software (GraphPad, San Diego, CA). Values of P<0.05 were considered significant.

## Results

### Induction of Distinct ppins-specific CD8 T-cells is Critically Dependent on the Antigen Used

**Figure 1:**
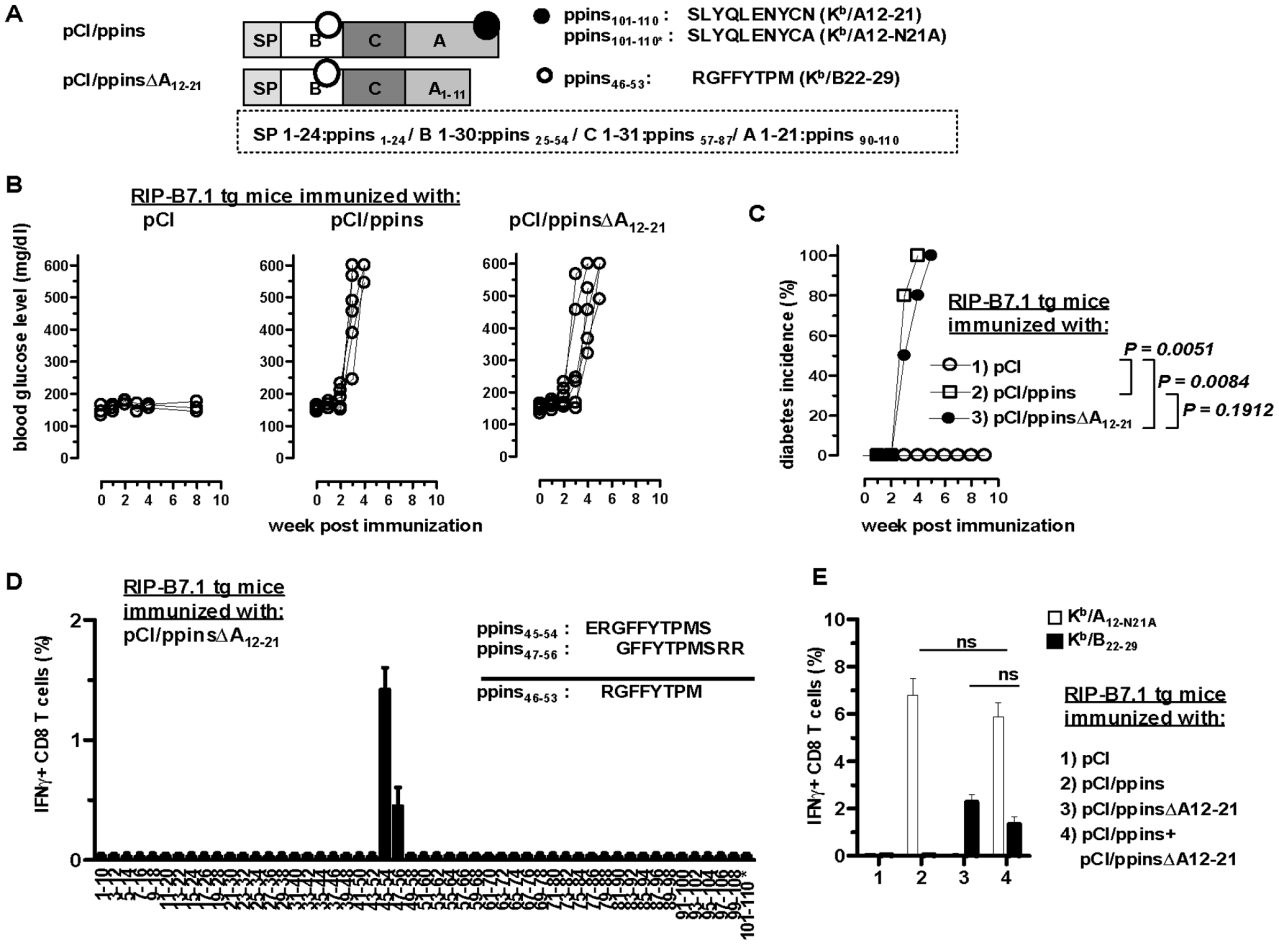
The RIP-B7.1 diabetes model. (**A**) Map of ppins antigens. The expression vectors encoding the ppins and the mutant ppinsΔA_12–21_ are shown. The signal peptide (SP), the insulin B- and A- chains, the C-peptide and the position and sequences of the K^b^/A_12–21_ epitope (•), its K^b^/A_12-N21A_ variant and of the newly identified K^b^/B_22–29_ epitope (○) are indicated. (**B,C**) RIP-B7.1 tg mice were immunized with pCI (groups 1, n = 6), pCI/ppins (groups 2, n = 6) or pCI/ppinsΔA_12–21_ (groups 3, n = 6). At indicated times after immunization, blood glucose levels (**B**) and cumulative diabetes incidences (**C**) were determined. The statistical significance of diabetes induction in immunized mice was determined using the log-rank test. Values of P<0.05 were considered significant. (**D**) CD8 T-cells were prepared from pancreata of pCI/ppinsΔA_12–21_-immune and diabetic RIP-B7.1 tg mice. Pancreatic cell preparations from ten mice were pooled and restimulated *ex vivo* for 16 hours with a ppins-specific peptide library (i.e., 10 mers with two amino acids offset) and frequencies of IFNγ^+^ CD8 T-cells were determined by by flow cytometry (FCM). The mean % of IFNγ^+^ CD8 T-cells in the pancreatic CD8 T-cell population (obtained from two independent experiments) are shown. CD8 T-cell frequencies <0.05% are defined negative. (**E**) RIP-B7.1 tg mice were immunized with pCI (group 1), pCI/ppins (group 2), pCI/ppinsΔA_12–21_ (group 3) or pCI/ppins and pCI/ppinsΔA_12–21_ (group 4). In group 4, the indicated plasmids were injected into the right and the left tibialis anterior muscles, respectively. CD8 T-cells were prepared from pancreata of diabetic (groups 2–4) or non-diabetic (group 1) mice and restimulated *ex vivo* with A_12-N21A_ or B_22–29_ peptides. Specific IFNγ^+^ CD8 T-cell frequencies were determined by FCM. The mean % of IFNγ^+^ CD8 T-cells in the pancreatic CD8 T-cell population (±SD) of a representative experiment (n = 3 mice per group) is shown. The statistical significance of differences between A_12-N21A_- (groups 2 and 4) and K^b^/B_22–29_-specific CD8 T-cell frequencies (groups 3 and 4) was determined by the unpaired Student’s t-test (ns, not significant).

A single injection of pCI/ppins plasmid DNA (Figure 1A) efficiently induced CD8 T-cell-mediated EAD in RIP-B7.1 tg mice (Figure 1B and C) [19]. CD8 T-cells isolated from pancreata of ppins-primed, diabetic RIP-B7.1 tg mice recognized the K^b^-restricted A_12–21_ (i.e., ppins_101–110_) epitope of ppins and, with a better efficacy, an epitope variant (A_12-N21A_) with an alanine (A) exchange for the COOH-terminal asparagine (N) at position A_21_ (Figure 1A) [19]. Similarly, a pCI/ppinsΔA_12–21_ DNA (encoding a truncated ppins protein without the COOH-terminal K^b^/A_12–21_ epitope; Figure 1A) also induced severe hyperglycemia and diabetes in RIP-B7.1 tg mice (Figure 1B and C) [18]. The kinetics and diabetes incidences were comparable in pCI/ppins- and pCI/ppinsΔA_12–21_-immune RIP-B7.1 tg mice (Figure 1B and C).

CD8 T-cells isolated from pancreata of pCI/ppinsΔA_12–21_-primed and diabetic RIP-B7.1 tg mice specifically recognized the overlapping ppins_45–54_ and ppins_47–56_ peptides of a ppins library (i.e., 10 mers with two amino acids offset; Figure 1D). These sequences contain an optimal K^b^-binding motif, *i.e.,* Y at anchor position P5 and M at anchor position P8 [33]. *Ex vivo* restimulation of CD8 T-cells with this antigenic ppins_46–53_ (B_22–29_) peptide revealed a CD8 T-cell population with specifically inducible IFNγ expression in pCI/ppinsΔA_12–21_- (but not in pCI/ppins-) immune and diabetic RIP-B7.1 tg mice (Figure 1E, groups 2 and 3; Table S1). We could exclude that a simple immune competition between K^b^/A_12–21_- and K^b^/B_22–29_-specific CD8 T-cells [34] limits the priming and expansion of K^b^/B_22–29_-specific CD8 T-cells in pCI/ppins-immune RIP-B7.1 tg mice. K^b^/A_12–21_- and K^b^/B_22–29_-specific CD8 T-cells were efficiently co-primed when pCI/ppins and pCI/ppinsΔA_12–21_ plasmids were co-injected into different sites of the same mouse (Figure 1E, group 4). Comparable numbers of K^b^/A_12-N21A_- and K^b^/B_22–29_-specific IFNγ^+^ CD8 T-cells were detectable in the pancreata of diabetic RIP-B7.1 tg mice immunized with pCI/ppins, pCI/ppinsΔA_12–21_ or both, pCI/ppins+pCI/ppinsΔA_12–21_ vectors, respectively (Figure 1E, groups 2–4; Table S1). Hence, after successful priming, the two CD8 T-cell populations do not interfere with each another. Furthermore, immunization of MHC class II-deficient (Aα^−/−^) RIP-B7.1 tg mice (RIP-B7.1^+^/MHC-II^−/−^) with pCI/ppins [18] and pCI/ppinsΔA_12–21_ (Figure S1) efficiently induced EAD. This showed that diabetogenic ppins/(K^b^/A_12–21_)- and ppinsΔA_12–21_/(K^b^/B_22–29_)-specific CD8 T-cell responses do not require CD4 T-cell help.

**Figure 2:**
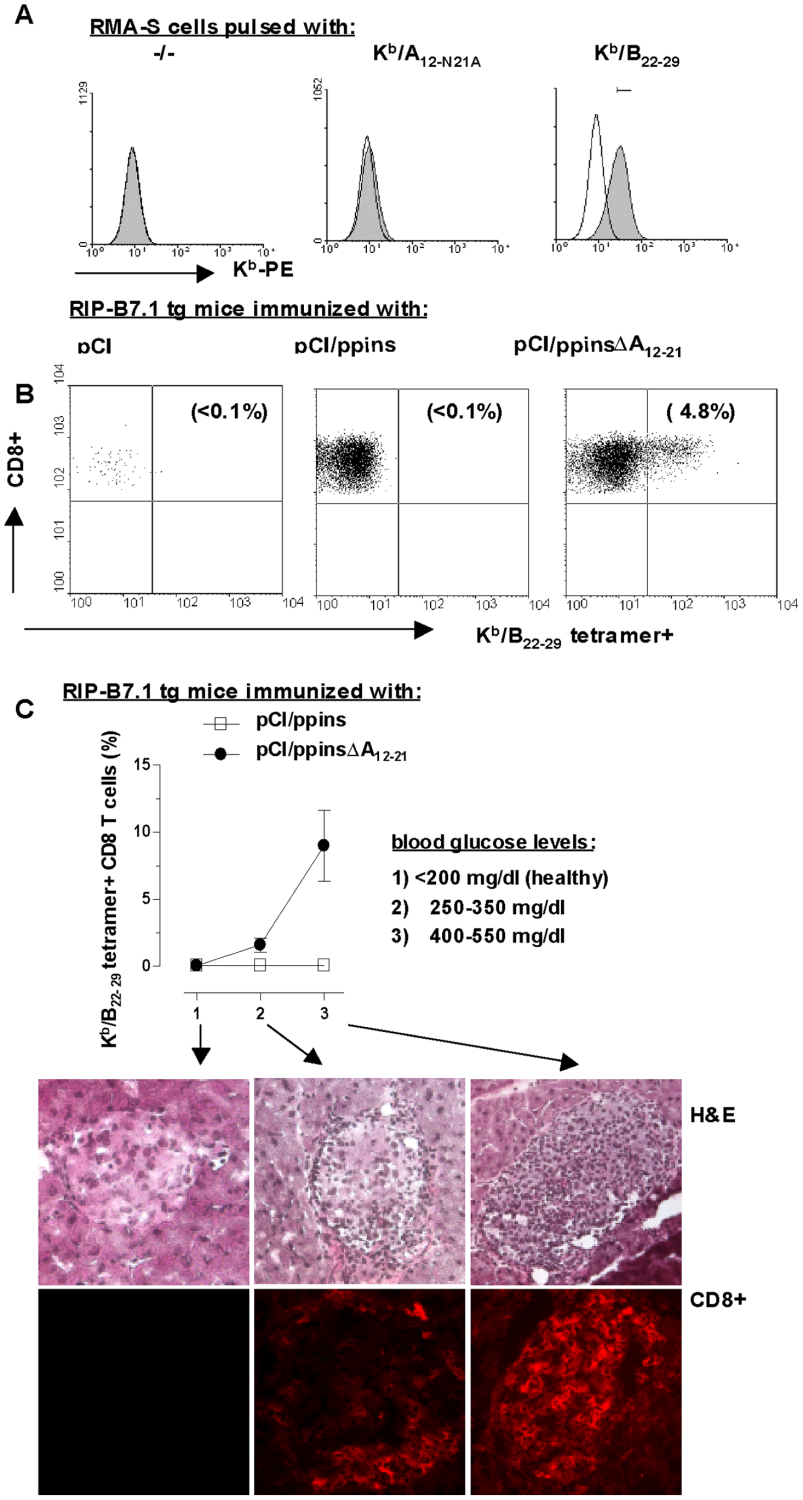
Determination of K^b^/B_22–29_-tetramer^+^ CD8 T-cells in diabetic RIP-B7.1 tg mice. (**A**) TAP-deficient RMA-S cells were either not pulsed (−/−) or pulsed for 6 h with high doses (100 µg/ml) of K^b^/A_12-N21A_ or K^b^/B_22–29_ peptides, followed by surface staining of trimeric K^b^-molecules and FCM. (**B**) RIP-B7.1 tg mice were immunized with pCI, pCI/ppins or pCI/ppinsΔA_12–21_. CD8 T-cells were prepared from pancreata of early diabetic (pCI/ppins, pCI/ppinsΔA_12–21_) or non-diabetic (pCI) mice and directly stained with K^b^/B_22–29_-tetramers. Primary FACS data are shown for representative mice. The actual percentage of K^b^/B_22–29_-tetramer^+^ CD8 T-cells within the pancreas-infiltrating CD8 T-cell population is shown in brackets. (**C**) The numbers of K^b^/B_22–29_-tetramer^+^ CD8 T-cells were determined during the course of pCI/ppinsΔA_12–21_-mediated EAD: group 1, health mice (n = 3) with blood glucose levels <200 mg/dl; group 2, early diabetic mice (n = 3) with blood glucose levels between 250–350 mg/dl; group 3, diabetic mice (n = 3) with severe diabetes (i.e., blood glucose levels between 400–550 mg/dl). Pancreata of representative mice out of groups 1 to 3 were analyzed histologically for CD8 T-cell influx (CD8+) or stained with hematoxylin-eosin (H&E).

The novel insulin B-chain epitope B_22–29_ efficiently stabilized the class I molecules K^b^ on the surface of TAP-deficient RMA-S cells (Figure 2A) [35]. The B_22–29_ epitope stabilized K^b^-molecules more efficiently than the A_12–21_ or mutant A_12-N21A_ epitopes (Figure 2A, data not shown) and we could generate K^b^/B_22–29_- (but neither K^b^/A_12–21-_ nor A_12-N21A-_) specific dimers or tetramers (Figure 2B; data not shown). K^b^/B_22–29_-tetramer^+^ CD8 T-cells were specifically detectable in pCI/ppinsΔA_12–21_- (but not in pCI/ppins-) primed and diabetic RIP-B7.1 tg mice (Figure 2B and C). During the course of EAD, the development of hyperglycemia correlated with an increasing influx of lymphocytes and CD8 T-cells into the pancreatic islets (Figure 2C, groups 1–3). In diabetic mice (with blood glucose levels between 400 and 550 mg/dl) 0.8–2×10^3^ K^b^/B_22–29_-tetramer^+^ CD8 T-cells were detectable in the pancreata. This corresponds to 7–12% of all pancreas-infiltrating CD8 T-cells (Figure 2C, group 3). The influx of K^b^/B_22–29_-specific CD8 T-cells into the pancreata thus specifically correlated with the development of disease in ppinsΔA_12–21_-immune RIP-B7.1 tg mice.

### Characterization of Antigen Expression Requirements that Favour Priming of K^b^/B_22–29_-Specific CD8 T-cells and EAD by DNA-based Immunization

**Figure 3:**
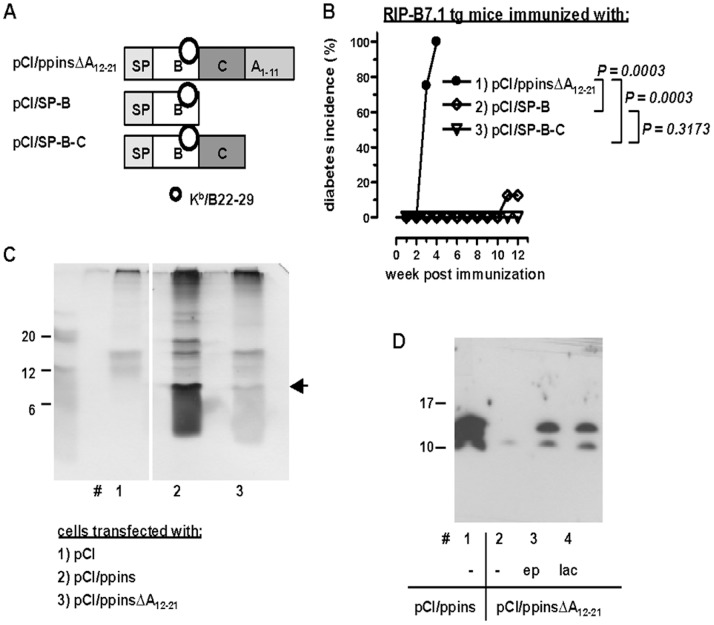
Priming of K^b^/B_22–29_-specific CD8 T-cell responses and EAD by mutant ppins antigens. (**A**) Map of the expression vectors pCI/ppinsΔA_12–21,_ pCI/SP-B (encoding the ER-targeting signal peptide and the insulin B-chain) and pCI/SP-B-C (encoding the ER-targeting signal peptide up to the C-peptide). The position of the K^b^/B_22–29_ epitope (○) is indicated. (**B**) RIP-B7.1 tg mice were immunized with pCI/ppinsΔA_12–21_ (group 1, n = 4), pCI/SP-B (group 2, n = 8) or pCI/SP-B-C DNA (group 3, n = 8) and cumulative diabetes incidences were determined. The statistical significance of diabetes induction in immunized mice was determined using the log-rank test. Values of P<0.05 were considered significant. (**C**) HEK-293 cells were transiently transfected with pCI (lane 1), pCI/ppins (lane 2) or pCI/ppinsΔA_12–21 _DNA (lane 3). Cells were labeled with ^35^S-methionine/cysteine, lysed and immunoprecipitated with an anti-insulin (H86) Ab and protein G sepharose. Immunoprecipitates were processed for SDS-PAGE, followed by fluorography of the gels. The position of pins is indicated (**D**) HEK-293 cells were transiently transfected with pCI/ppins (lane 1) or pCI/ppinsΔA_12–21_ (lanes 2–4). At 28 h after transfection, cells were either non-treated (lanes 1 and 2), or incubated for 6 h with the proteasome-inhibitors expoxymycin (ep; lane 3) or lactacystein (lac; lane 4) and subsequently lysed. Total cell extracts were subjected to high resolution tricine-urea-SDS-PAGE (16%) followed by anti-insulin (H86) specific western blotting.

The efficient induction of K^b^/B_22–29_-specific CD8 T-cells and EAD by mutant ppinsΔA_12–21_ was unexpected because immunization with different insulin B-chain-encoding vectors did not (or very inefficiently) induce EAD in RIP-B7.1 tg mice. A pCI/SP-B construct (encoding the ER-targeting signal peptide and the insulin B-chain; Figure 3A), inefficiently induced late EAD in one out of eight RIP-B7.1 tg mice (Figure 3B, group 2). Similarly, a pCI/SP-B-C construct (encoding the ER-targeting signal peptide up to the C-peptide; Figure 3A) did not induce EAD in RIP-B7.1 tg mice within three months post immunization (Figure 3B, group 3). We thus conclude that efficient priming of K^b^/B_22–29_-reactive CD8 T-cells by pCI/ppinsΔA_12–21_ critically depends on specific properties of the mutant antigen itself.

We next characterized the expression of mutant ppinsΔA_12–21_ and ppins in transiently transfected HEK-293 cells (Figure 3C and D). In these non-beta cells, the ppins signal peptide (SP) targets the proteins into the ER, where the SP is removed to generate proinsulin (pins) or pinsΔA_12–21_ but further downstream processing of pins to insulin was not detectable [17]; [36]. The expression levels of ^35^S-methionine/cysteine labeled pinsΔA_12–21_ were weaker than that of pins (Figure 3C, lanes 2 and 3) and significant steady-state levels of pins (but not of mutant pinsΔA_12–21_) were detectable by specific western blot analyses (Figure 3D, lanes 1 and 2). However, treatment of transfectants with the proteasome inhibitors epoxomicin or lactacystin efficiently restored pinsΔA_12–21_ levels within 6 hours (Figure 3D, lanes 2 to 4). This showed that the pinsΔA_12–21_ is efficiently processed by proteasomal degradation. In contrast, the expression of ppins in transiently transfected HEK-293 cells was not changed by proteasome inhibitors [18]. This implies that proteasomes play an essential role in the pCI/ppinsΔA_12–21_-specific antigen processing/presentation and the induction of K^b^/B_22–29_ specific CD8 T-cells.

### Differential Regulation of Diabetogenic K^b^/A_12–21_- and K^b^/B_22–29_-specific CD8 T-cell Responses in Coinhibition-deficient PD-L1^−/−^ Mice

**Figure 4:**
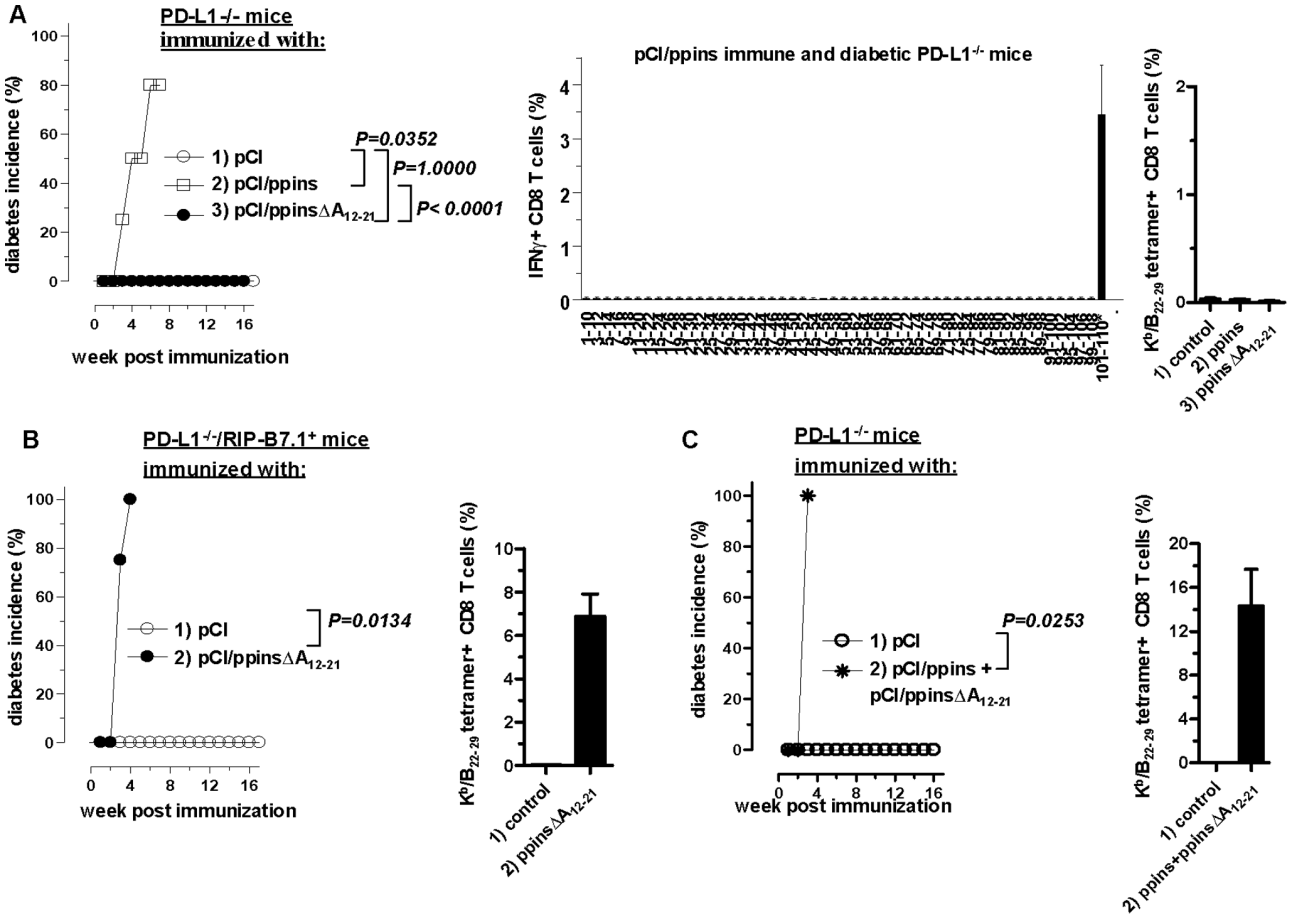
Characterization of autoreactive CD8 T-cell responses in PD-L1^−/−^ mice. (**A**) PD-L1^−/−^ mice were immunized with pCI (group 1, n = 3), pCI/ppins (group 2, n = 10) or pCI/ppinsΔA_12–21_ (group 3, n = 20) and cumulative diabetes incidences (%) were determined (left panel). CD8 T-cells were prepared from pancreata of pCI/ppins-immune and diabetic PD-L1^−/−^ mice. Pancreatic cell preparations from eight ppins-immune mice were pooled and restimulated *ex vivo* for 16 hours with a ppins-specific peptide library (i.e., 10 mers with two amino acids offset) and frequencies of IFNγ^+^ CD8 T-cells were determined by FCM. The mean % of IFNγ^+^ CD8 T-cells in the pancreatic CD8 T-cell population (obtained from two independent experiments) are shown (middle panel). Pancreatic cell preparations from ppins-immune and diabetic (group 2, n = 3), and from control (pCI) or ppinsΔA_12–21_-immune and healthy PD-L1^−/−^ mice (groups 1 and 3, n = 3) were directly stained with K^b^/B_22–29_-tetramers. The percentage of K^b^/B_22–29_-tetramer^+^ CD8 T-cells (±SD) within the pancreas-infiltrating CD8 T-cell population is shown (right panel). (**B**) RIP-B7.1^+^/PD-L1^−/−^ mice were immunized with pCI (group 1, n = 3) or pCI/ppinsΔA_12–21_ (group 2, n = 4) and cumulative diabetes incidences (%) (left panel) and K^b^/B_22–29_-tetramer^+^ CD8 T-cells in the pancreata (right panel) were determined as described above. (**C**) PD-L1^−/−^ mice were immunized with pCI (group 1, n = 2) or both, pCI/ppins+pCI/ppinsΔA_12–21_ vectors (group 2, n = 4) into the right and the left tibialis anterior muscles, respectively and cumulative diabetes incidences (%) (left panel) and K^b^/B_22–29_-tetramer^+^ CD8 T-cells in the pancreata (right panel) were determined as described above. The statistical significance of diabetes induction in immunized mice (**A–C**) was determined using the log-rank test. Values of P<0.05 were considered significant.

Coinhibitory interactions of PD-1 (expressed on T-cells) with PD-L1 (expressed on APCs) inhibit T-cell activation and promote induction of peripheral T-cell tolerance [23]; [25]. We used coinhibition-deficient PD-L1^−/−^ [29] and PD-1^−/−^ [30] mice to determine whether EAD is equally induced by ppins- and ppinsΔA_12–21_-specific CD8 T-cells. Immunization of PD-L1^−/−^ mice with pCI/ppins efficiently induced CD8 T-cell-mediated EAD (Figure 4A, left panel, group 2) [19]. Comparable with RIP-B7.1 tg mice, a K^b^/A_12–21_-monospecific CD8 T-cell response was detectable in pCI/ppins-primed and diabetic PD-L1^−/−^ mice (Figure 4A, middle panel), and K^b^/B_22–29_-specific tetramer^+^ CD8 T-cells were not detectable (Figure 4A, right panel, group 2). Unexpectedly, PD-L1^−/−^ mice did not develop EAD after single or repeated immunizations with pCI/ppinsΔA_12–21_ (Figure 4A, left panel, group 3; data not shown). We could neither detect T-cell infiltrations into the pancreatic islets (Figure S2A) nor K^b^/B_22–29_-specific CD8 T-cells in these healthy mice (Figure 4A, right panel, group 3; Table S1). We could not induce EAD in PD-L1^−/−^ mice after immunization with pCI/ppinsΔA_12–21_ and acutely depletion of regulatory CD25^+^ CD4^+^ T-cells (T_reg_) by anti CD25 antibody treatment (data not shown) [37]. It is thus unlikely that T_reg_ cells inhibit the outcome of K^b^/B_22–29_-specific CD8 T-cells in ppinsΔA_12–21_-immune PD-L1^−/−^ mice. Similarly, coinhibition-deficient PD-1^−/−^ mice efficiently developed EAD after immunization with pCI/ppins [19] but not pCI/ppinsΔA_12–21_ (Figure S3). An imbalance between PD-1/PD-L1-interactions thus facilitated development of EAD by pCI/ppins/(K^b^/A_12–21_)- but not pCI/ppinsΔA_12–21_/(K^b^/B_22–29_)-specific CD8 T-cells.

K^b^/B_22–29_-specific CD8 T-cells were either inefficiently primed in PD-L1^−/−^ and PD-1^−/−^ mice by pCI/ppinsΔA_12–21_ and/or inefficiently expanded and targeted to the pancreatic islets. We next generated PD-L1-deficient mice, which selectively express the costimulatory B7.1 molecule on beta cells (RIP-B7.1^+^/PD-L1^−/−^) by crossing PD-L1^−/−^ with RIP-B7.1 tg mice. These mice differ from PD-L1^−/−^ mice only in the tg B7.1 expression in beta cells. Interestingly, immunization of RIP-B7.1^+^/PD-L1^−/−^ mice with pCI/ppinsΔA_12–21_ efficiently induced EAD and high frequencies of K^b^/B_22–29_-specific CD8 T-cells accumulated in the pancreata of diabetic mice (Figure 4B, groups 2). Notably, pCI/ppins/(K^b^/A_12–21_)-specific CD8 T-cells efficiently induced EAD in both, PD-L1^−/−^ and RIP-B7.1^+^/PD-L1^−/−^ mice (Table S1) [19]. K^b^/B_22–29_- (but not K^b^/A_12–21_-) specific CD8 T-cells thus require B7.1-mediated costimulatory signals from PD-L1-deficient beta cells to expand and/or develop their diabetogenic potential.

### A Concomitant ppins/(K^b^/A_12–21_)-specific CD8 T-cell Response in PD-L1^−/−^ Mice Facilitates Induction of ppinsΔA_12–21_/(K^b^/B_22–29_)-specific CD8 T-cells

**Figure 5:**
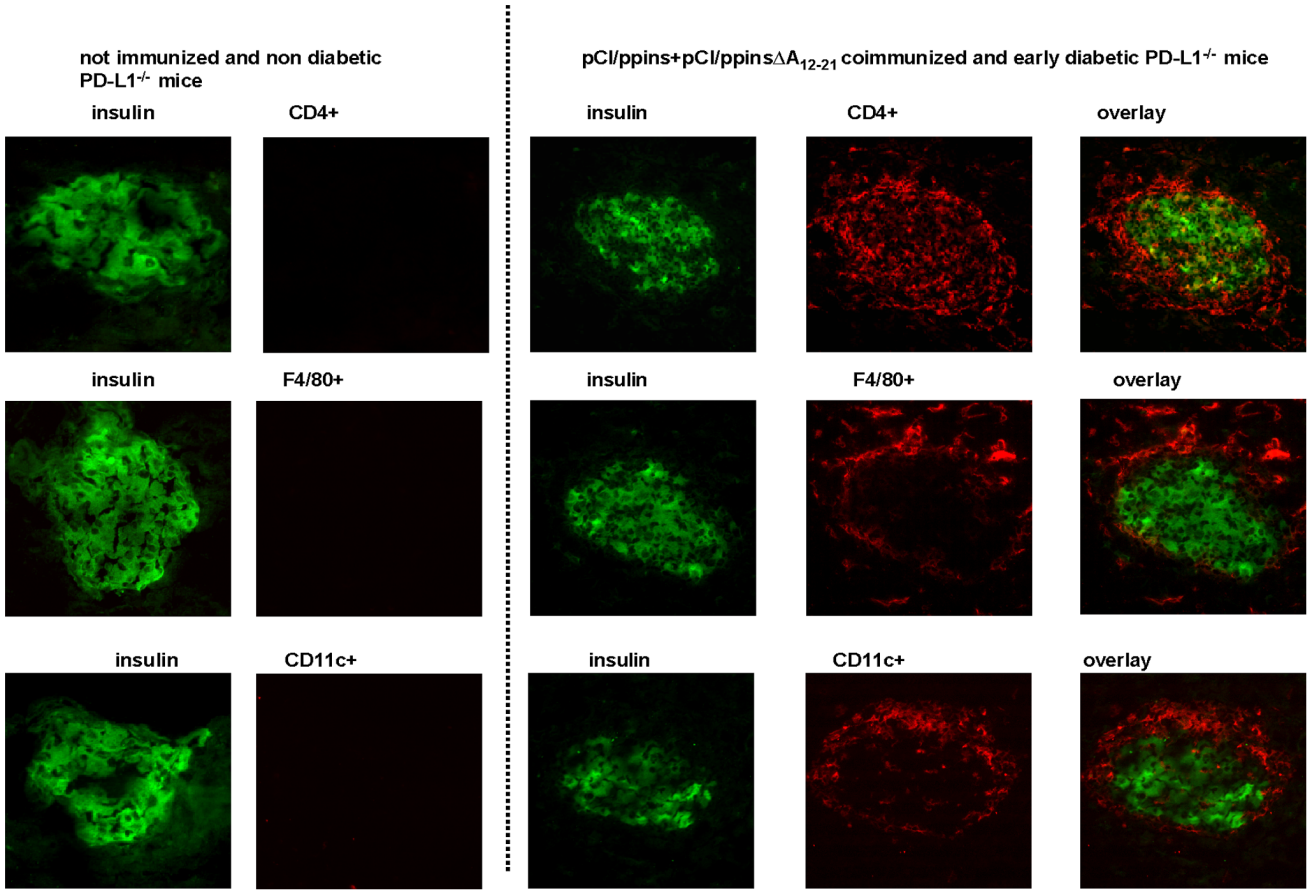
Recruitment of different ‘bystander’ cell populations into the pancreatic target tissue. PD-L1^−/−^ mice were immunized with both, pCI/ppins+pCI/ppinsΔA_12–21_ vectors into the right and the left tibialis anterior muscles, respectively. Pancreata of representative healthy (at 3 days post immunization) (A) or early diabetic mice (at 15–20 days post immunization) (B) were analyzed histologically for insulin expression (insulin) and influx of CD4^+^ T-cells (CD4^+^), macrophages (F4/80^+^) or DCs (CD11c^+^).

We next asked whether alternative (B7.1 tg-independent) beta cell-mediated signals could trigger the diabetogenic ppinsΔA_12–21_/(K^b^/B_22–29_)-specific CD8 T-cell response in PD-L1^−/−^ mice. It was well established that an initial damage or destruction of beta cells by autoreactive T-cells induces a complex inflammatory milieu in the islets, thereby attracting different non-specific “bystander” cells [38], [39], [40]. These events play a prominent role in the amplification of autoreactive immune responses and beta cell destruction [38], [39], [40]. We thought that an initial islet-destructive K^b^/A_12–21_-specific CD8 T-cell response (primed in PD-L1^−/−^ mice by pCI/ppins) could facilitate the expansion of pCI/ppinsΔA_12–21_-coprimed K^b^/B_22–29_-specific CD8 T-cells. To test this assumption, we co-immunized PD-L1^−/−^ mice with both, pCI/ppins and pCI/ppinsΔA_12–21_ vectors (pCI/ppins+pCI/ppinsΔA_12–21_) into the left and right tibialis anterior muscles, respectively. These mice efficiently developed an early and severe EAD (Figure 4C, left panel, group 2). Comparable with pCI/ppins-immune and diabetic PD-L1^−/−^ mice, high numbers of K^b^/A_12–21_-specific CD8 T-cells were detectable in pCI/ppins+pCI/ppinsΔA_12–21_-coimmunized and diabetic PD-L1^−/−^ mice (see Figure 4A; data not shown). In these mice, we found a significant influx of CD8 T-cells (Figure S2B) and other bystander cells (e.g., CD4 T-cells, macrophages, DCs) into or closely attached to the pancreatic islets of early diabetic PD-L1^−/−^ mice (Figure 5). The inflammatory islet invasion by these cell populations was comparable in pCI/ppins- and pCI/ppins+pCI/ppinsΔA_12–21_-immune and diabetic (but not in pCI/ppinsΔA_12–21_-immune and healthy) PD-L1^−/−^ mice (data not shown). Most interestingly, high numbers of K^b^/B_22–29_-specific CD8 T-cells accumulated in the pancreata of pCI/ppins+pCI/ppinsΔA_12–21_-coimmunized PD-L1^−/−^ mice (Figure 4C, right panel, group 2). Because K^b^/B_22–29_-specific CD8 T-cells were detectable in pCI/ppins+pCI/ppinsΔA_12–21_ co-immunized PD-L1^−/−^ mice, but not in PD-L1^−/−^ mice injected with the individual ppins- and ppinsΔA_12–21_-encoding vectors (Figure 4A and C; Table S1), their expansion was apparently induced by events triggered by the initial ppins/(K^b^/A_12–21_)-specific CD8 T-cell response.

## Discussion

We continued work on the specific priming of ppins-specific CD8 T-cells and EAD in RIP-B7.1 tg mice [16], [17], [18], [19]. DNA-based immunization of RIP-B7.1 tg mice revealed two monospecific CD8 T-cell responses that were exclusively induced by either pCI/ppins (primes K^b^/A_12–21_-specific CD8 T-cells) or pCI/ppinsΔA_12–21_ (primes K^b^/B_22–29_-specific CD8 T-cells). This indicated that the mutant ppinsΔA_12–21_ antigen (but not the ppins) efficiently induced K^b^/B_22–29_-specific CD8 T-cells in RIP-B7.1 tg mice. We further characterized the antigen expression requirements that favour *in vivo* priming of K^b^/B_22–29_-specific CD8 T-cells and EAD by DNA-based immunization. Different insulin B-chain-encoding vectors (pCI/SP-B or pCI/SP-B-C) did not or very inefficiently induce K^b^/B_22–29_-specific CD8 T-cells and EAD in RIP-B7.1 tg mice (Figure 3A and B). Deletion of the A_12–21_ sequence may thus generate a specifically folded ppinsΔA_12–21_ antigen, which is efficiently processed for K^b^/B_22–29_-specific epitope presentation. Expression analyses in transiently transfected HEK-293 cells showed that ppinsΔA_12–21_ (but not ppins) is efficiently processed by proteasomes, resulting in a high turnover expression of this mutant antigen. Proteasomes could thus play an essential role in the generation/presentation of the K^b^/B_22–29_ epitope and the induction of K^b^/B_22–29_-specific CD8 T-cells by pCI/ppinsΔA_12–21_.

We here showed that K^b^/B_22–29_-specific CD8 T-cells are efficiently primed by ppinsΔA_12–21_- (but not ppins)-expressing vectors. This indicates that ppins is inefficiently processed for K^b^/B_22–29_ epitope presentation in non-beta cells targeted by intramuscular DNA injection. However, the K^b^/B_22–29_ epitope is efficiently processed and presented *in vivo* by ppins/insulin-expressing beta cells because ppinsΔA_12–21_/(K^b^/B_22–29_)-monospecific CD8 T-cells specifically recognize and destroy these cells and induce fulminant EAD in RIP-B7.1 tg mice (Figure 1). The efficient beta cell-specific presentation of the K^b^/B_22–29_ epitope could be explained by different antigen expression and/or processing mechanisms, operating in ppins/insulin producing beta cells and in ppins-expressing non-beta APCs [36]. However, further studies are needed to define the inefficient processing of ppins in non-beta cells and/or the inefficient induction of K^b^/B_22–29_-specific CD8 T-cells by pCI/ppins.

We consider the differential regulation of K^b^/A_12–21_- and K^b^/B_22–29_-monospecific CD8 T-cell responses (and EAD) by costimulatory and coinhibitory signals the key observation of this report. K^b^/A_12–21_-monospecific CD8 T-cells and EAD were efficiently induced in RIP-B7.1 tg and coinhibition-deficient PD-L1^−/−^ or PD-1^−/−^ mice by pCI/ppins, whereas K^b^/B_22–29_-specific CD8 T-cells and EAD were efficiently induced in RIP-B7.1 tg (but not in PD-L1^−/−^ or PD-1^−/−^) mice by pCI/ppinsΔA_12–21_. The missing coinhibition in PD-L1^−/−^ or PD-1^−/−^ mice is thus sufficient to induce and expand vector-primed K^b^/A_12–21_- (but not K^b^/B_22–29_)- specific CD8 T-cells. PD-L1 expressed by antigen presenting beta cells can interact with PD-1 or B7.1 expressed by CD8 T-cells to inhibit immune responses [25], [41]. Interestingly, expression of PD-L1 has no impact on the priming of K^b^/A_12–21_-specific CD8 T-cells in PD-L1-competent wt B6 mice. However, ppins-immune B6 mice rapidly developed EAD after treatment with anti PD-L1 antibody [19]. This suggested that PD-L1-mediated signals delivered by pancreatic beta cells are sufficient to regulate their susceptibility for the destructive K^b^/A_12–21_-specific CD8 T-cell attack [19].

We found no evidence for an autoreactive immune response in pCI/ppinsΔA_12–21_-immune and healthy PD-L1^−/−^ or PD-1^−/−^ mice. Interestingly, a single manipulation of the PD-L1 mouse model (i.e., the tg expression of the costimulatory B7.1 molecule in beta cells) restored the induction of K^b^/B_22–29_-specific CD8 T-cells and EAD in these RIP-B7.1^+^/PD-L1^−/−^ mice by pCI/ppinsΔA_12–21_. It is thus unlikely that the initial CD8 T-cell priming phase (i.e., intramuscular injection of pCI/ppinsΔA_12–21_ DNA; local antigen expression/processing and K^b^/B_22–29_-specific epitope presentation in PD-L1-deficient myocytes and professional APCs; priming of PD-L1-deficient K^b^/B_22–29_-specific CD8 T-cells) differs in RIP-B7.1^+^/PD-L1^−/−^ and PD-L1^−/−^ mice. Expression of the tg B7.1 costimulator molecule by PD-L1-deficient beta cells is thus a key event that decides whether a K^b^/B_22–29_-specific T-cell response can progress and develop a functional pathogenic phenotype. This implies that primed K^b^/B_22–29_-specific CD8 T-cells must directly interact with RIP-B7.1^+^ beta cells to expand and/or develop their diabetogenic potential. B7.1 on the surface of beta cells could bind in trans to CD28 costimulator molecule or CTLA-4/PD-L1 coinhibitor molecules on the surface of T-cells or in cis to PD-L1 expressed by beta cells [21]. CD28-deficient RIP-B7.1^+^/CD28^−/−^ mice do not develop EAD after immunization with pCI/ppins [19] or pCI/ppinsΔA_12–21_ (data not shown). The interaction of B7.1 on the surface of beta cells with CD28 costimulator molecules on CD8 T-cells may thus promote T-cell-driven EAD in RIP-B7.1 tg mice by facilitating effector function delivery but a critical effect of CD28 in CD8 T-cell priming can not be excluded.

We here identified an alternative mechanism to promote the expansion and influx of diabetogenic K^b^/B_22–29_-specific CD8 T-cells into the pancreata of pCI/ppinsΔA_12–21_-primed and diabetic PD-L1^−/−^ mice. Co-immunization of PD-L1^−/−^ mice with both, pCI/ppins+pCI/ppinsΔA_12–21_ vectors (but not with the individual pCI/ppins or pCI/ppinsΔA_12–21_ vectors) efficiently elicited both, K^b^/A_12–21_- and K^b^/B_22–29_-specific CD8 T-cells (Table S1). This suggested that the initial beta cell destruction, triggered by pCI/ppins/(K^b^/A_12–21_)-specific CD8 T-cells in PD-L1^−/−^ mice, facilitates expansion and invasion of K^b^/B_22–29_-specific CD8 T-cells but also other bystander cells into the pancreatic target tissue. The specific molecular mechanisms and signals expanding and attracting K^b^/B_22–29_-specific CD8 T-cells to the pancreas and the role of bystander cells are not well understood [38], [39], [40]. An initial beta cell death and antigen release could facilitate activation of circulating, pCI/ppinsΔA_12–21_-preprimed K^b^/B_22–29_-specific CD8 T-cells in the regional lymph nodes by professional APCs [42]. Furthermore, an altered local cytokine milieu and expression of cell surface receptors [40] or an enhanced antigen presentation by beta cells [43] in inflamed islets may favour the attraction and/or activation of K^b^/B_22–29_-specific CD8 T-cells. Taken together, our findings suggested that the K^b^/A_12–21_-specific CD8 T-cell response directly initiates beta cell destruction in PD-L1^−/−^ mice, whereas a downstream K^b^/B_22–29_-specific CD8 T-cell response requires additional activation signals *in vivo* and emerge during the pathogenic destruction of beta cells. Interestingly, distinct hierarchies of diabetogenic T-cell responses were also detectable in the NOD mouse model. The insulin B9–23 domain, containing both, a dominant CD4 and a K^d^-restricted B_15–23_ CD8 T-cell epitope, plays a prominent role in the diabetes development in NOD mice [10], [44], [45]. Prasad et al. previously showed that an initial insulin B9–23-specific T-cell response is immunodominant and autoimmune responses to epitope(s) distinct from B9–23 emerge during the pathogenic progression of diabetes in NOD mice [46]. Similarly, CD8 T-cells specific for the islet-specific glucose-6-phosphatase catalytic subunit-related protein (IGRP) were detected in NOD mice but not in tg NOD mice tolerant to proinsulin, indicating that an initial T-cell response against proinsulin is necessary for the development of IGRP-specific CD8 T-cells [47]. This suggests that distinct CD8 T-cell responses, triggering the initial steps of beta cell destruction, play a prime role in the induction of diabetes [44].

In conclusion, we showed in this study that inhibitory interactions between ppins/insulin-presenting beta cells and autoreactive CD8 T-cells either allow or prevent activation of pathogenic effector responses. K^b^/B_22–29_-reactive CD8 T-cells additionally require stimulatory signals from beta cells or inflamed islets to expand and develop their diabetogenic potential in PD-L1-deficient mice. In contrast, CD8 T-cells directed against the weak K^b^/A_12–21_ epitope (binding K^b^ molecules with a relatively low avidity as compared with the K^b^/B_22–29_ epitope; see Figure 2A) do not depend on the tg B7.1-mediated help to reveal their diabetogenic potential in ppins-immune PD-L1^−/−^ or PD-1^−/−^ mice. Differences in the MHC-binding avidity of ppins-derived epitopes may thus have a strong impact on the regulation of autoreactive CD8 T-cell responses in PD-1- or PD-L1-deficient mice. The novel PD-1/PD-L1 diabetes models are thus valuable tools to study under well controlled experimental conditions the induction and regulation of autoreactive CD8 T-cell responses.

## Supporting Information

Figure S1 **Induction of EAD in MHC II-deficient RIP-B7.1 tg mice.** MHC class II-deficient RIP-B7.1 tg mice (RIP-B7.1^+^/MHCII^−/−^) were immunized with pCI (group 1, n = 3) or pCI/ppinsΔA_12–21_ (group 2, n = 3). At indicated times after immunization, blood glucose levels and cumulative diabetes incidences were determined.
(EPS)

Figure S2 **Ppins/(K^b^/A_12–21_)-mediated recruitment of autoreactive T-cells into the pancreatic target tissue.** PD-L1^−/−^ mice were immunized with pCI/ppinsΔA_12–21_ (**A**) or with both, pCI/ppins+pCI/ppinsΔA_12–21_ vectors into the right and the left tibialis anterior muscles, respectively (**B**). Pancreata of representative healthy (**A**) and early diabetic mice (**B**) were analyzed histologically for insulin expression (insulin) and influx of CD8^+^ T-cells (CD8^+^), or stained with hematoxylin-eosin (H&E).
(TIF)

Figure S3 **Induction of CD8 T-cell-mediated EAD in PD-1^−/−^ mice.** PD-1^−/−^ mice were immunized with pCI (group 1, n = 3), pCI/ppins (group 2, n = 6) or pCI/ppinsΔA_12–21_ (group 3, n = 12) and cumulative diabetes incidences (%) were determined.
(EPS)

Table S1 **Induction of autoreactive CD8 T-cell responses and EAD in RIP-B7.1^+^**
(DOC)
