# Supplementary figures and images for: A Missing PD-L1/PD-1 Coinhibition Regulates Diabetes Induction by Preproinsulin-Specific CD8 T-Cells in an Epitope-Specific Manner
Source: PLoS One. 2013 Aug 19;8(8):e71746. doi: 10.1371/journal.pone.0071746 (PMC3747217; doi:10.1371/journal.pone.0071746)

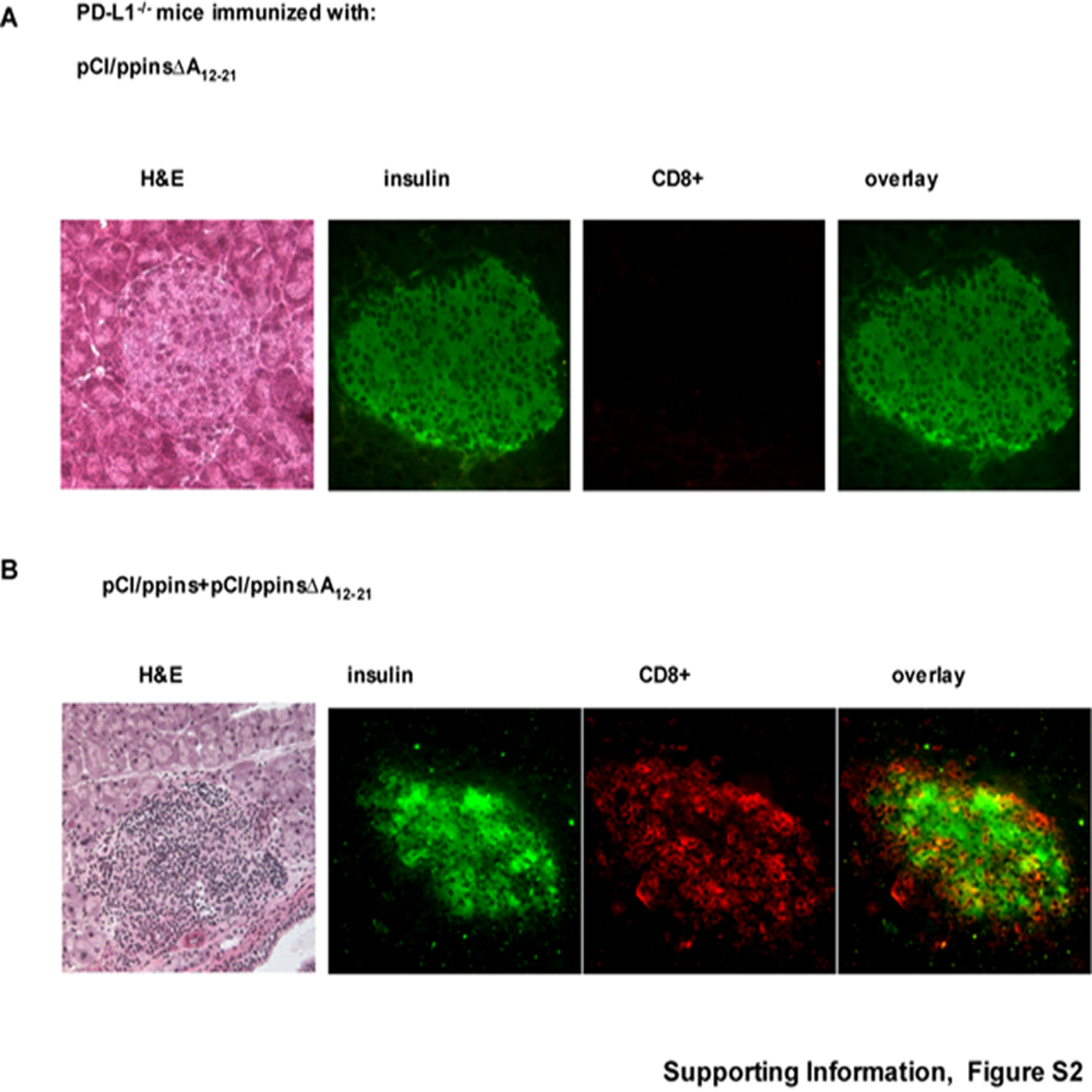

Supplement: Figure S2 — Ppins/(Kb/A12–21)-mediated recruitment of autoreactive T-cells into the pancreatic target tissue. PD-L1−/− mice were immunized with pCI/ppinsΔA12–21 (A) or with both, pCI/ppins+pCI/ppinsΔA12–21 vectors into the right and the left tibialis anterior muscles, respectively (B). Pancreata of representative healthy (A) and early diabetic mice (B) were analyzed histologically for insulin expression (insulin) and influx of CD8+ T-cells (CD8+), or stained with hematoxylin-eosin (H&E). (TIF) [file pone.0071746.s002.tif]
