# Supplementary material for: A Missing PD-L1/PD-1 Coinhibition Regulates Diabetes Induction by Preproinsulin-Specific CD8 T-Cells in an Epitope-Specific Manner
Source: PLoS One. 2013 Aug 19;8(8):e71746. doi: 10.1371/journal.pone.0071746 (PMC3747217; doi:10.1371/journal.pone.0071746)
Supplement: Table S1 — Induction of autoreactive CD8 T-cell responses and EAD in RIP-B7.1+ (DOC) [file pone.0071746.s004.doc]

**Table S1**

**Induction of autoreactive CD8 T-cell responses and EAD in RIP-B7.1^+^**

**and PD-L1-deficient mice.**

| **mice** | **antigen** | **K^b^/A_12-21_** | **K^b^/B_22-29_** | **EAD** |
| --- | --- | --- | --- | --- |
| RIP-B7.1 | ppins | 🗸 | _ | + |
| PD-L1^-/-^ | ppins | 🗸 | _ | + |
| RIP-B7.1^+^/PD-L1^-/-^ | ppins | 🗸 | _ | + |
|  |  |  |  |  |
| RIP-B7.1 | ppinsΔA_12-21_ | _ | 🗸 | + |
| PD-L1^-/-^ | ppinsΔA_12-21_ | _ | _ | _ |
| RIP-B7.1^+^/PD-L1^-/-^ | ppinsΔA_12-21_ | _ | 🗸 | + |
|  |  |  |  |  |
| RIP-B7.1 | ppins + ppinsΔA_12-21_ | 🗸 | 🗸 | + |
| PD-L1^-/-^ | ppins + ppinsΔA_12-21_ | 🗸 | 🗸 | + |

RIP-B7.1, PD-L1^-/-^ and RIP-B7.1^+^/PD-L1^-/-^ mice were immunized with pCI/ppins, pCI/ppinsΔA_12-21_ or both, pCI/ppins+pCI/ppinsΔA_12-21_ vectors. K^b^/A_12-21_- and K^b^/B_22-29_-specific CD8 T-cells were determined in the pancreata of healthy (EAD -) and diabetic (EAD +) mice as described in Figs. 1, 2 and 4. CD8 T-cells were detectable (🗸) or not detectable (-).
